# Takayasu Arteritis Masquerading as Shoulder Pain: A Case Report

**DOI:** 10.31729/jnma.7443

**Published:** 2022-04-30

**Authors:** Prabhat Poudel, Paras Oli, Prashant Ojha, Radhay Shyam Yadav, Binaya Raj Poudel

**Affiliations:** 1 Nepal Medical College Teaching Hospital, Attarkhel, Kathmandu, Nepal; 2 Department of Nephrology, Institute of Medicine, Maharajgunj, Kathmandu, Nepal; 3 Department of Internal Medicine, Nepal Medical College Teaching Hospital, Attarkhel, Kathmandu, Nepal

**Keywords:** *arteritis*, *inflammation*, *shoulder pain*, *takayasu arteritis*

## Abstract

Takayasu arteritis is a rare progressive chronic granular inflammation of the vessels that mainly affects the aorta and its branches. It is widely distributed in the world population and mainly involves young Asian women, manifesting as a systemic illness with myriads of cardiovascular signs and symptoms. The current case focuses on a young girl who had shoulder pain and weakness as the only manifestation of underlying Takayasu arteritis. Early clinical identification of the disease and control with disease-modifying anti-rheumatic drugs could improve the outcome and prevent devastating sequelae.

## INTRODUCTION

Takayasu arteritis is a rare progressive chronic granular inflammation of the vessels that mainly affects the aorta and its branches.^1^ The most common affected population is women in the second to third decades. Common clinical symptoms include hypertension, heart failure, impaired renal function, cerebral vascular disease, paralysis, depression, and headache.^2^ Clinically, it is diagnosed by a history of claudication, absence of pulse, blood pressure fluctuations, aortic noise, and specific angiographic observations. In this paper, we report the case of a 17-year-old girl complaining of long-term shoulder pain, as a manifestation of Takayasu arteritis.

## CASE REPORT

A 17-year-old girl from rural Nepal started developing dull aching pain in her left shoulder joint lasting about eight months. Before coming to our hospital, she was receiving Non-steroidal Anti-Inflammatory Drugs (NSAIDs) for joint pain that was unable to provide any symptomatic relief. Her pain started insidiously and progressed to involve other shoulder and bilateral knees. The pain was very severe during the evenings and prevented her from performing her daily activities. Upon further questioning, she also complained of a generalised weakness that was insidious on the onset, apparently starting as easy fatigability during sports play and progressing to difficulty in attending her school and finally unable to do basic chores. She also complains about loosening her clothes, although she did not track her weight before visiting us. Her weight was 36 kilograms. She had no history of fever, night sweats, blurred vision, bruises, dizziness, headache, nausea, vomiting, or sore throat.

On physical examination, the patient appeared to be ill and wasted. There was a remarkable blood pressure discrepancy between the right and left arms. Blood pressure was undetectable in the left arm, but 100/50 mm Hg supine in the right arm. The pulse was 98 beats per minute with radio-radial delay, and pulse volume was more on the right side than on the left side. All the peripheral pulses were palpable. On auscultation, the heart sounds were clear with a panwsystolic murmur in the mitral area, louder on expiration and radiating to the axilla also there was an audible bruit on bilateral carotids, bilateral subclavian artery, abdominal aorta, and renal arteries, all of which were louder on the right side.

We performed an examination of her bilateral shoulders that revealed no localised tenderness, no difference in the temperature of the right and left shoulders, and no limitations in the range of motion of either shoulder joint.

The electrocardiography revealed no more than sinus tachycardia and echocardiography revealed a dilated left ventricle with an ejection fraction of 30% and a mild mitral regurgitation. Contrast-Enhanced Computed Tomography (CECT) showed homogeneous enhanced circumferential, regular and long segment thickening of bilateral common carotid arteries, left subclavian artery, arch of the aorta, descending thoracic and abdominal aorta. There was minimally thickened and homogeneous enhancement of the main pulmonary trunk and bilateral pulmonary arteries. Saccular dilatation of the proximal part of ascending aorta, proximal part of the descending aorta, and the proximal part of the abdominal aorta. These radiological findings were suggestive of type V takayasu arteritis. She had a Magnetic Resonance Imaging (MRI) of her shoulder joint that was done at another centre which revealed no abnormalities.

Ultrasonography (USG) Doppler study of the abdominal aorta and renal arteries revealed diffuse wall thickening of the abdominal aorta and bilateral renal arteries, post stenotic turbulent flow in the abdominal aorta, increased peak systolic velocity in bilateral renal arteries, and increased Renal Aortic Ratio (RAR).

Erythrocyte Sedimentation Rate (ESR) was 115 mm in the first hour and C-Reactive Protein (CRP) was 97 mg/l. We used the criteria of the American College of Rheumatology (ACR 1990) and criteria established by Kerr GS, et al. at the national institute of health to make the diagnosis. The Indian Takayasu Clinical Activity Score (ITAS) 2010 score was calculated to be 14 on the first day and remained the same before discharge. Rheumatoid Arthritis (RA) factor assay was within normal range and Antinuclear Antibodies (ANA) were negative which helped to rule out Systemic Lupus Erythematosus (SLE).

She was treated in the line of Takayasu arteritis with oral prednisolone and azathioprine with a proton pump inhibitor and supplemental calcium and she is visiting us regularly for monitoring her disease status and adjusting the therapy with a satisfactory outcome.

## DISCUSSION

Takayasu arteritis is a chronic progressive necrotizing granulomatous panvasculitis of large vessels that mainly includes the aorta and its main branches.^1,3^ Takayasu Arteritis was first described in 1830 by the Japanese Oriental medicine practitioner Rokushu Yamamoto. Among the first scientific presentations of this disease, Mikito Takayasu presented it in 1905 as the case of a 21-year-old woman with a peculiar coronary anastomosis in her optic plexus.^4^ In 1990, it was included on the list of intractable diseases of the Japanese government.^5^ Takayasu arteritis is widely distributed throughout the world but is more common among Asians. In Japan, the incidence of TA is estimated at 1 to 2 per million, and in Kuwait 2.2 per million it was found to be highest in Japan (40 per million) and lowest in the United States (0.9 per million).^6^

Systemic inflammation symptoms such as weight loss, fatigue, fever, fatigue, arthritic, and myalgia are common at the beginning of the disease. Our patient has been suffering from fever, weight loss, depression, and arthritis for eight months. Arterial inflammation can also cause headache, thoracic pain, and carotidynia, and if the inflammation is severe enough to cause arterial compromise, it can result in limb claudication, abdominal angina, cardiac angina, heart failure, dizziness, and neurovascular manifestations such as transient ischemic attacks and strokes.^7^

In a study of 272 patients with Takayasu arteritis, hypertension was found to be the most common clinical characteristic and occurs in 77% of cases.^8^ Our patient had an average blood pressure of 90/50 mmHg during a hospital stay. Hypertension that is very common in patients with Takayasu arteritis could be explained by stenosis of the aorta and renal artery; both of them were present in our patient. On echocardiography, our patient had left heart failure with an ejection fraction of 30% which could explain her low blood pressure despite the stenosis of the aorta and bilateral renal arteries. Cardiac failure was the most common problem encountered at the first presentation of the disease.

The diagnosis of Takayasu arteritis can be made based on the criteria of the American College of Rheumatology (ACR 1990) criteria.^9^ A criteria modified from ACR 1990 criteria for the diagnosis of Takayasu arteritis was used.^10^ In addition to the obligatory criterion, the presence of two major criteria, one major and two or more minor criteria, or four more minor criteria suggests a high probability of the presence of Takayasu's disease.

The disease activity can be accessed by a criteria established at the National Institutes of Health,^10^ according to which, new-onset or worsening of two or more of the features indicates active disease. Our patient met the obligatory criteria, 1 major (left mid-subclavian artery) and 6 minor (ACR 1990 criteria for the diagnosis of Takayasu arteritis), and meets 4/4 of the criteria for disease activity.^11^

Glucocorticoids are the main components of medical therapy, and recent literature suggests that the initial dose of prednisolone is low, at 0.5 mg/kg/day, and can be used to control the activity of the disease.^12^ The use of conventional Disease Modifying Anti Rheumatic Drugs (DMARDs), such as methotrexate, azathioprine, Mycophenolate Mofetil (MMF), leflunomide, and cyclophosphamide, can be helpful as a steroid-sparing agent and can also be useful to maintain long-term remission. Amongst all the DMARDs, MMF appears to be the most promising, as it is safe and highly effective in controlling disease activity in Takayasu arteritis.^13^

In conclusion, autoimmune vasculitis of this nature could be masquerading under clinical presentation of joint pain and some constitutional symptoms; early recognition and management of such could improve the quality of life of the patient and prevent life-threatening consequences.
